# Burden of Comorbidities and Healthcare Resource Utilization Among Medicaid-Enrolled Extremely Premature Infants

**DOI:** 10.36469/001c.38847

**Published:** 2022-12-23

**Authors:** Meredith E. Mowitz, Wei Gao, Heather Sipsma, Pete Zuckerman, Hallee Wong, Rajeev Ayyagari, Sujata P. Sarda

**Affiliations:** 1 Division of Neonatology, University of Florida, Gainesville; 2 Analysis Group, Inc., Boston, Massachusetts; 3 Global Evidence and Outcomes, Takeda Pharmaceutical Company Limited, Lexington, Massachusetts

**Keywords:** extreme prematurity, healthcare resource utilization, Medicaid, bronchopulmonary dysplasia, chronic lung disease

## Abstract

**Background:** The effect of gestational age (GA) on comorbidity prevalence, healthcare resource utilization (HCRU), and all-cause costs is significant for extremely premature (EP) infants in the United States.

**Objectives:** To characterize real-world patient characteristics, prevalence of comorbidities, rates of HCRU, and direct healthcare charges and societal costs among premature infants in US Medicaid programs, with respect to GA and the presence of respiratory comorbidities.

**Methods:** Using *International Classification of Diseases, Ninth/Tenth Revision, Clinical Modification* codes, diagnosis and medical claims data from 6 state Medicaid databases (1997-2018) of infants born at less than 37 weeks of GA (wGA) were collected retrospectively. Data from the index date (birth) up to 2 years corrected age or death, stratified by GA (EP, ≤28 wGA; very premature [VP], >28 to <32 wGA; and moderate to late premature [M-LP], ≥32 to <37 wGA), were compared using unadjusted and adjusted generalized linear models.

**Results:** Among 25 573 premature infants (46.1% female; 4462 [17.4%] EP; 2904 [11.4%] VP; 18 207 [71.2%] M-LP), comorbidity prevalence, HCRU, and all-cause costs increased with decreasing GA and were highest for EP. Total healthcare charges, excluding index hospitalization and all-cause societal costs (US dollars), were 2 to 3 times higher for EP than for M-LP (EP 74 436vsM−LP27 541 and EP 28 504vsM−LP15 892, respectively).

**Conclusions:** Complications of preterm birth, including prevalence of comorbidities, HCRU, and costs, increased with decreasing GA and were highest among EP infants during the first 2 years in this US analysis.

## BACKGROUND

Globally, around 15 million births each year are preterm, occurring before 37 weeks gestation.^1,2^ Extremely premature (EP) births—those occurring at less than 28 weeks of gestational age (wGA)—account for approximately 6% of total preterm births.^3^ Preterm births represent a major challenge in the field of obstetrics and neonatology,^1,4^ owing to an increased risk of long-term respiratory, neuropsychiatric, and cardiovascular pathologies.^5–8^ These precipitously early deliveries disrupt lung development, which can result in short- and long-term respiratory complications.^7^ In premature infants, risks for morbidity and mortality increase proportionally with decreasing gestational age (GA).^9^ Extremely premature infants are at high risk of complications such as intraventricular hemorrhage (IVH), retinopathy of prematurity (ROP), and chronic lung disease including bronchopulmonary dysplasia (BPD), which often result in mortality or long-term disabilities.^8,10–13^

The management of premature infants with complications incurs high utilization of healthcare resources and a high cost burden.^14–29^ Medical costs (adjusted to 2015 US values) for the birth hospitalization of infants delivered at 24 wGA have been reported to range between US dollars (USD) $111 152 and USD $576 972, compared with the range between USD $930 and USD $7114 for full-term infants, and an inverse relationship between GA at birth and overall costs has been reported.^26^ Extremely premature infants discharged with respiratory complications require a high level of respiratory support and continue to need complex care regimens at home.^30^ These infants, if they survive to adulthood, are also predisposed to chronic respiratory diseases such as asthma and chronic obstructive pulmonary disease,^31^ both of which incur long-term direct and societal costs.^21^

Despite improved survival of EP infants in recent years, there remains a significant risk of developing short- and long-term complications.^32–35^ Current options are limited for effectively treating neonates with complications associated with prematurity. Administered prenatally, maternal betamethasone can be used to stimulate lung maturity and magnesium sulfate can be used to reduce cerebral palsy.^36,37^ Although ventilators and surfactant therapy increase survival rates in preterm infants with respiratory distress syndrome (RDS), they can contribute to pneumonia, sepsis, pneumothorax, pulmonary hemorrhage, and IVH.^38^ Less-invasive surfactant therapies and oxygen titration have been developed to reduce rates of BPD and ROP, and continuous positive airway pressure oxygenation is used to reduce rates of ROP, RDS, and BPD.^39–41^ Despite these advances, the most common complication affecting EP infants is BPD, which continues to impact respiratory function into adulthood.^7^

The high costs associated with preterm birth have been documented previously, including costs of initial hospitalizations^18,25,28^ and incremental costs per comorbidity,^15^ costs of resource use in early childhood and through to adulthood,^23,26,27,42^ societal costs for families of premature infants,^16,29,42^ and annual costs relative to near-term and full-term infants.^19,23,29^ All previous analyses that factored in GA and/or birthweight found that the highest costs are associated with the earliest preterm births. In a Canadian study through age 10 years, costs were higher for infants born early preterm compared with infants born moderate or late preterm.^42^ A similar trend was reported in a study to age 18 years in England and Wales.^23^

Studies report increased costs throughout early childhood for children delivered at less than 37 wGA, primarily due to increased resource use, including rehospitalizations and doctor visits. Through discharge to age 6 years, the odds ratio for hospitalization was 1.3-1.6 compared with children delivered at full term.^27^ In an analysis of all premature infants born 1996-1997 in Quebec, Ontario, Canada, Johnston et al^42^ found that costs in Canadian dollars (CAD) through age 10 years were $67 467 for early preterm infants and CAD $52 796 for moderate preterm infants vs CAD $10 010 for late preterm infants. Mangham et al^23^ reported a similar trend, extending the estimate to age 18 years.

A national cost estimate in Canada from 1996 found that although EP births accounted for only 6.7% of preterm births, they accounted for 21.0% of the total national cost of preterm births.^42^ Hall and Greenberg^19^ reported significant cost reductions for initial hospitalization, as well as community-level costs, that would result from an increase in GA by 1-week increments.

There remains a clear unmet need for therapies and clinical practices that reduce the prevalence of complications of prematurity and their associated longer-term morbidity burden, especially in EP infants. Determination of the major drivers of costs and healthcare resource utilization (HCRU) associated with premature infants, in both the short and long term, will help inform practitioners and policy makers. This study aimed to characterize real-world patient characteristics, prevalence of comorbidities, rates of HCRU, and direct healthcare charges and societal costs among premature infants in US Medicaid programs with respect to GA and the presence of respiratory comorbidities.

## METHODS

### Study Design

Premature infants were identified retrospectively from approximately 26.6 million Medicaid beneficiaries from 6 US state Medicaid databases that were available from 1997 to 2018 (Iowa, Kansas, New Jersey, Mississippi, Missouri, and Wisconsin) and followed for up to 2 years of corrected age (CA). Patients were identified by GA using the *International Classification of Diseases, Ninth/Tenth Revision, Clinical Modification* (ICD-9-CM/ICD-10-CM) codes (**Supplementary Table S1**). Inclusion criteria included premature infants (<37 wGA) who were born during the data availability period; infants admitted to the intensive care unit (ICU) or neonatal ICU (NICU) within 1 day after birth, where the first hospitalization with ICU/NICU admission is defined as the index (birth) hospitalization; and infants with continuous eligibility from birth to 2 years CA or before death, where CA was defined as:

CA in Years−[(40 Weeks−GA in Weeks)×7/365]

Infants were excluded if they died before reaching 36 weeks postmenstrual age, or if they were diagnosed with congenital heart disease, diaphragmatic hernia, or other major congenital malformations from birth to death, 1 year CA, or end of continuous eligibility, whichever occurred first.

The database contained data related to patient demographic characteristics, enrollment history, and complete medical and pharmaceutical claims from Medicaid records for all eligible patients (including Medicare/Medicaid crossovers), identified by codes listed in **Supplementary Tables S2-S9**. The first NICU/ICU admission was defined as the index hospitalization. Bronchopulmonary dysplasia was defined as at least 1 diagnosis code of BPD in the ICD-9-CM/ICD-10-CM, during the period from birth to death or end of continuous eligibility (**Supplementary Table S7**). Chronic lung disease, determined for the EP cohort only, was defined as infants with at least 1 of the following events in 2 consecutive quarters during 1 year CA, excluding the index hospitalization: use of home oxygen therapy or respiratory aids, use of respiratory medications, or presence of respiratory symptoms (cough and wheeze) (**Supplementary Tables S5 and S6**).

Overall HCRU, direct charges, and societal costs were calculated from claims records. Subsequently, the numbers of events per person-year (PPY) and counts and percentages of patients with any use in each utilization category were estimated. Healthcare charge adjustments were made by the health plan and were not part of this analysis. The charges given in the claims data were used and adjusted to 2018 USD using the US Medical Services Component of the Consumer Price Index.

For direct charges, the majority of claims were capitation claims, which are fixed advance payments adjusted for each patient based on age and health status, paid monthly by the health plan directly to service providers irrespective of actual services received (eg, the number of visits a patient had). Because the amount paid for a capitation claim does not reflect the costs of the actual services provided, healthcare charges were analyzed individually for each claim.

Because this was a retrospective cohort analysis of existing de-identified patient data and did not involve experiments on humans or the use of human tissues/samples, patient consent and approval from an institutional review board were exempt, per Title 45 CFR § 46.101.^43^ Analysis Group implemented all safeguards that were required for the protection of the individuals whose data were included in the study. All methods were carried out in accordance with relevant guidelines and regulations.

### Statistical Analyses

Descriptive statistics, including counts and percentages for categorical variables, and mean and SD for continuous variables, were generated. Unadjusted and adjusted generalized linear models with a negative binomial distribution and a log link were used to compare the number of HCRU events and length of stay between GA cohorts up to 2 years CA or death, and models with a Tweedie distribution and a log link were used to compare charges and costs between cohorts. Unadjusted and adjusted logistic regression models were used to compare the presence of HCRU events between cohorts. Adjusted models comparing GA cohorts accounted for demographic characteristics (sex, state of birth, length of index hospitalization, cesarean vs vaginal delivery, birth year, and multiple birth) and comorbidities (BPD, IVH, ROP, periventricular leukomalacia [PVL], necrotizing enterocolitis, bacterial sepsis, and spontaneous intestinal perforation).

Incidence rates of rehospitalizations, emergency department (ED) visits, and outpatient visits after the index hospitalization due to respiratory causes and all causes were calculated by GA up to 2 years CA or death.

Societal costs were estimated for each individual patient by multiplying the average hourly wage (calculated from state-specific average hourly wages from the 2017 US Census data, inflated to 2018 USD) by the hours of work loss incurred for 1 parent: work loss was assumed to be 2 hours for each outpatient visit, 4 hours for each ED visit, and 8 hours for each day in the hospital.

Data were reported by GA cohort: EP (≤28 wGA), very premature (VP) (>28 to <32 wGA), and moderate to late premature (M-LP) (≥32 to <37 wGA) infants. Note that although the World Health Organization definition of “extremely preterm” denotes infants less than 28 wGA, infants born at 28 weeks cannot be separated out owing to a limitation of the ICD-9-CM code, specifically, the ICD-9-CM code 765.24, which includes infants born at 27 to 28 completed gestation weeks; thus, the definition of EP for this study includes infants born up to 28 weeks 6 days GA.

## RESULTS

### Patient Characteristics

**Figure 1.**
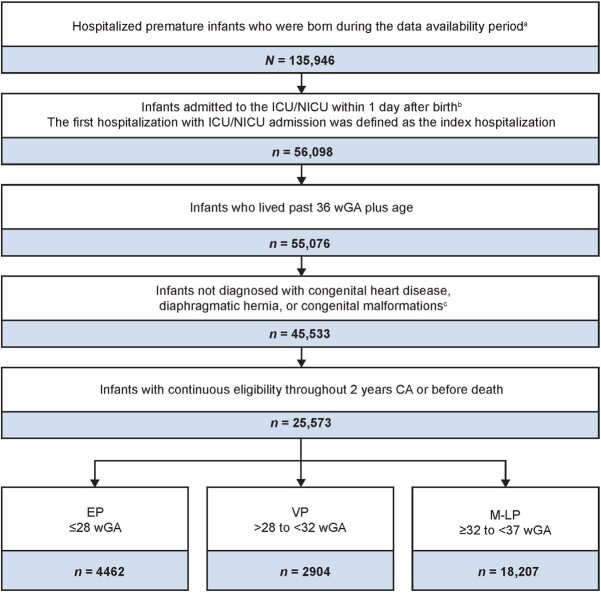
Sample Selection of Analysis Population and Gestational Age Cohorts Abbreviations: CA, corrected age; EP, extremely premature; ICU, intensive care unit; ICD-9-CM/ICD-10-CM, *International Classification of Diseases, Ninth/Tenth Revision, Clinical Modification*; M-LP, moderate to late premature; NICU, neonatal intensive care unit; VP, very premature; wGA, weeks of gestational age. ^a^Infants born at less than 37 wGA were identified using ICD-9-CM/ICD-10-CM codes from the US Medicaid claims database (see **Supplementary Table S1**). ^b^Admission to ICUs/NICUs were identified using hospital standard revenue codes (see **Supplementary Table S2**). Patients with an index hospitalization greater than 365 days were excluded. ^c^Congenital heart disease, diaphragmatic hernia, and congenital malformation were identified using ICD-9-CM and ICD-10-CM codes (see **Supplementary Table S3**) during the period from birth to death, 1 year CA, or end of continuous eligibility, whichever happened first. Infants with a diagnosis of patent ductus arteriosus or patent foramen ovale were not excluded.

From 1997 to 2018, a total of 25 573 premature infants (46.1% female) were included in the study; 4462 (17.4%) were EP (≤28 wGA), 2904 (11.4%) were VP, and 18 207 (71.2%) were M-LP (Figure 1). Characteristics and demographics during the index hospitalization are shown in Table 1. Sex distributions were similar between GA cohorts. Among race categories, Black infants comprised 32.5%, 28.3%, and 20.1% of EP, VP, and M-LP infants, respectively, while White infants comprised 38.0%, 42.2%, and 46.0% of EP, VP, and M-LP infants, respectively. Index hospitalization length of stay (LOS) increased with decreasing GA: EP infants had a mean LOS of 83.24 days compared with 48.82 days among infants with VP and 20.02 days among infants with M-LP. Follow-up duration was similar across the 3 cohorts.

**Table 1.** Patient Characteristics/Demographics During Birth Hospitalization by Gestational Age Cohort

**Characteristic/Demographic**	**EP Infants (≤28 wGA) (n = 4462)**	**VP Infants (>28 to <32 wGA) (n = 2904)**	**M-LP Infants (≥32 to <37 wGA) (n = 18 207)**
Female, n (%)	2218 (49.7)	1344 (46.3)	8215 (45.1)
Race, n (%)			
White	1694 (38.0)	1225 (42.2)	8369 (46.0)
Black	1450 (32.5)	823 (28.3)	3656 (20.1)
Other/multiracial	476 (10.7)	327 (11.3)	2206 (12.1)
Not reported	842 (18.9)	529 (18.2)	3976 (21.8)
State, n (%)			
Iowa	830 (18.6)	657 (22.6)	6416 (35.2)
Kansas	568 (12.7)	411 (14.2)	2785 (15.3)
Mississippi	654 (14.7)	492 (16.9)	2340 (12.9)
Missouri	1283 (28.8)	798 (27.5)	3689 (20.3)
New Jersey	479 (10.7)	199 (6.9)	1175 (6.5)
Wisconsin	648 (14.5)	347 (11.9)	1802 (9.9)
Mean ± SD birth hospitalization LOS (days)	83.24 ± 36.92	48.82 ± 18.69	20.02 ± 16.62
Multiple birth, n (%)	870 (19.5)	671 (23.1)	3847 (21.1)
Delivery method, n (%)^a^			
Cesarean	2648 (59.3)	1826 (62.9)	9577 (52.6)
Vaginal	1468 (32.9)	958 (33.0)	8174 (44.9)
Not reported	346 (7.8)	120 (4.1)	456 (2.5)
Mean ± SD follow-up duration (days)	725.92 (107.63)	745.34 (71.18)	744.73 (67.90)

Abbreviations: EP, extremely premature; GA, gestational age; LOS, length of stay; M-LP, moderate to late premature; VP, very premature; wGA, weeks of gestational age.

^a^Identified using *International Classification of Diseases, Ninth/Tenth Revision, Clinical Modification* codes (**Supplementary Table S9**).

### Comorbidities

The prevalence of comorbidities during the birth hospitalization increased with decreasing GA (Table 2). In the EP cohort, the most prevalent comorbidities were acute RDS (91.5%), BPD (50.6%), and patent ductus arteriosus (PDA) (47.1%).Intubation for BPD/RDS was required for 54.1% of EP infants. Intraventribular hemorrhage occurred in 25.1% of infants, ROP in 22.3%, and PVL in 3.8%. Chronic lung disease affected 72.1% of EP infants. Further stratification for chronic lung disease revealed 78.0% (160/205) of infants born at 22 to 23 wGA, 74.0% (1488/2011) born at 24 to 26 wGA, and 69.9% (1569/2246) born at 27 to 28 wGA were affected.

**Table 2.** Occurrence of Comorbidities by Gestational Age Cohort During the Birth Hospitalization, n (%)

**Comorbidity**	**EP Infants (≤28 wGA) (n=4462)**	**VP Infants (>28 to <32 wGA) (n=2904)**	**M-LP Infants (≥32 to <37 wGA) (n=18 207)**
Cardiovascular			
PDA	2103 (47.1)	555 (19.1)	823 (4.5)
Gastrointestinal			
NEC^a^	463 (10.4)	128 (4.4)	239 (1.3)
Stage 1	28 (0.6)	11 (0.4)	18 (0.1)
Stage 2	44 (1.0)	18 (0.6)	30 (0.2)
Stage 3	61 (1.4)	7 (0.2)	9 (0.05)
Unspecified stage	137 (3.1)	26 (0.9)	66 (0.4)
Spontaneous intestinal perforation	97 (2.2)	11 (0.4)	32 (0.2)
Neurological^b^			
IVH	1121 (25.1)	416 (14.3)	593 (3.3)
Stage 1	344 (7.7)	244 (8.4)	373 (2.0)
Stage 2	226 (5.1)	56 (1.9)	58 (0.3)
Stage 3	159 (3.6)	39 (1.3)	38 (0.2)
Stage 4	224 (5.0)	24 (0.8)	17 (0.1)
Unspecified stage	159 (3.6)	51 (1.8)	104 (0.6)
Intracerebral hemorrhage	493 (11.0)	138 (4.8)	451 (2.5)
General neurological dysfunction	287 (6.4)	107 (3.7)	396 (2.2)
PVL	168 (3.8)	53 (1.8)	45 (0.2)
Cerebral palsy	3 (0.1)	0 (0)	4 (0.02)
Ophthalmological			
ROP	996 (22.3)	297 (10.2)	383 (2.1)
Stage 1	286 (6.4)	67 (2.3)	18 (0.1)
Stage 2	290 (6.5)	35 (1.2)	12 (0.1)
Stage 3	174 (3.9)	12 (0.4)	9 (0.05)
Stage 4	2 (0.04)	0 (0.0)	0 (0.0)
Stage 5	2 (0.04)	0 (0.0)	0 (0.0)
Unspecified stage	242 (5.4)	183 (6.3)	344 (1.9)
Retinal detachment	30 (0.7)	0 (0.0)	0 (0.0)
Other			
BPD	2256 (50.6)	418 (14.4)	437 (2.4)
Acute RDS	4082 (91.5)	2384 (82.1)	8242 (45.3)
Bacterial sepsis	180 (4.0)	67 (2.3)	166 (0.9)
Hearing loss	41 (0.9)	23 (0.8)	121 (0.7)
Bacterial meningitis	40 (0.9)	5 (0.2)	11 (0.1)
Medical procedures			
Intubation for BPD/RDS	2413 (54.1)	1115 (38.4)	2823 (15.5)
Ligation for PDA	406 (9.1)	22 (0.8)	9 (0.05)
Laser surgery/cryotherapy for ROP	110 (2.5)	2 (0.1)	1 (0.005)
Bowel surgery for NEC	92 (2.1)	15 (0.5)	29 (0.2)
Hydrocephalus shunting for IVH	107 (2.4)	21 (0.7)	14 (0.1)
Tracheostomy for BPD/RDS	28 (0.6)	1 (0.03)	12 (0.1)

Abbreviations: BPD, bronchopulmonary dysplasia; EP, extremely premature; GA, gestational age; ICD, *International Classification of Diseases*; ICD-9-CM, ICD, *Ninth Revision, Clinical Modification*; ICD-10-CM, ICD, *Tenth Revision, Clinical Modification*; IVH, intraventricular hemorrhage; M-LP, moderate to late premature; NEC, necrotizing enterocolitis; PDA, patent ductus arteriosus; PVL, periventricular leukomalacia; RDS, respiratory distress syndrome; ROP, retinopathy of prematurity; VP, very premature; wGA, weeks of gestational age.

^a^Stages for NEC do not equal total NEC because there are missing data due to incomplete codes; patients categorized as having NEC were not further categorized according to stage (1, 2, 3, or unspecified).

^b^IVH and intracerebral hemorrhage were defined with different sets of ICD codes, as follows, and do not overlap, although they may occur in the same patient: IVH, ICD-9-CM code 772.1, and ICD-10-CM codes P52.0–P52.3; intracerebral hemorrhage, ICD-9-CM codes 431 and 767, and ICD-10-CM codes I61 and P52.4. Codes for all comorbidities are listed in **Supplementary Table S7**. Codes for medical procedures are listed in **Supplementary Table S8.**

In the VP and M-LP cohorts, RDS was also the most prevalent comorbidity; however, a smaller percentage of infants were affected (82.1% and 45.3% of infants, respectively, compared with 91.5% in the EP group). In these cohorts, PDA was present in 19.1% and 4.5% of infants, BPD in 14.4% and 2.4%, IVH in 14.3% and 3.3%, ROP in 10.2% and 2.1%, and PVL in 1.8% and 0.2%, respectively.

### Healthcare Resource Utilization

The proportions of infants with rehospitalizations, ED visits, and outpatient visits from index hospitalization to 2 years CA or death were higher among EP infants than infants born at more than 28 wGA. All-cause rehospitalizations (≥1 per infant) were reported for 2001 (44.8%) EP infants, 984 (33.9%) VP infants, and 4534 (24.9%) ML-P infants. All-cause ED visits were reported for 3391 (76.0%) EP infants, 2130 (73.3%) VP infants, and 12 882 (70.8%) M-LP infants. All-cause outpatient visits were reported for 4206 (94.3%) EP infants, 2723 (93.8%) VP infants, and 15 780 (86.7%) ML-P infants.

**Figure 2.**
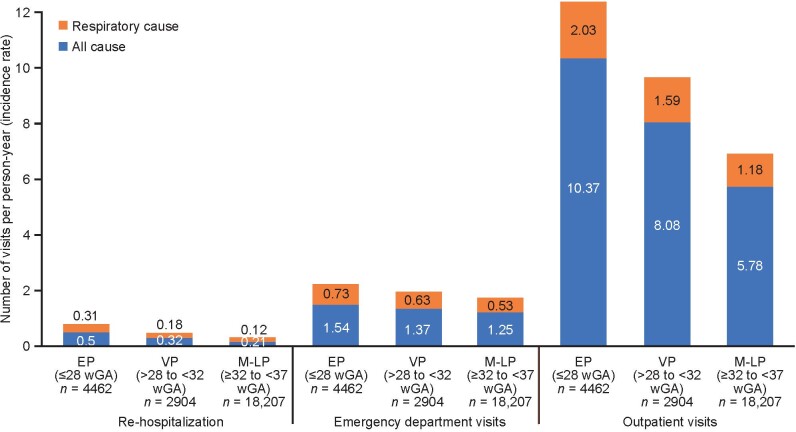
Incidence Rate of Rehospitalizations, Emergency Department Visits, and Outpatient Visits by Gestational Age Cohort, From Discharge of the Birth Hospitalization to 2 Years Corrected Age or Death Abbreviations: EP, extremely premature; M-LP, moderate to late premature; VP, very premature; wGA, weeks of gestational age. Respiratory causes of hospitalization, emergency department visits, and outpatient visits were identified using ICD-9-CM and ICD-10-CD codes (**Supplementary Table S4**). Results presented are observed/unadjusted.

Observed incidence rates of rehospitalizations were 0.50, 0.32, and 0.21 PPY for EP, VP, and M-LP infants, respectively; 1.54, 1.37, and 1.25 PPY for ED visits; and 10.37, 8.08, and 5.78 PPY for outpatient visits (Figure 2). Of the total number of rehospitalizations and visits, respiratory causes were responsible for 70.7% of rehospitalizations, 70.6% of ED visits, and 69.2% of outpatient visits, respectively.

Length of stay for all rehospitalizations was higher for infants with lower GA. The mean ± SD LOS for rehospitalizations for any cause was 22.53 ± 46.36 days for EP infants, 14.90 ± 32.81 days for VP infants, and 12.44 ± 34.59 days for M-LP infants. The mean ± SD LOS for rehospitalizations for respiratory causes was 19.46 ± 42.46 days for EP infants, 12.23 ± 22.29 days for VP infants, and 10.48 ± 25.65 days for M-LP infants.

### Direct Charges and Societal Costs

**Figure 3.**
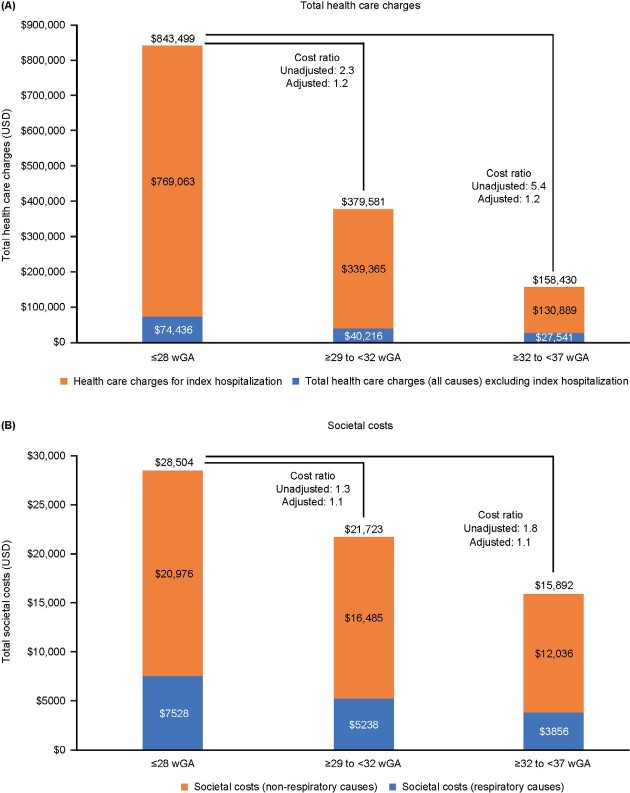
Total Healthcare Charges (**A**) and Total Societal Costs (**B**) by Gestational Age Cohort, From Birth Hospitalization to 2 Years Corrected Age or Death Abbreviations: USD, US dollars; wGA, weeks of gestational age.

Total healthcare charges were higher among EP infants compared with infants born at >28 wGA; total all-cause healthcare charges in USD, inflated to 2018 values, were $843 499, $379 581, and $158 430 for EP, VP, and M-LP infants, respectively (Figure 3A). Excluding birth hospitalization, all-cause charges were USD $74 436, $40 216, and $27 541, respectively, and respiratory-related charges were USD $39 231, $20 087, and $11 987, respectively.

Compared with the EP cohort, all-cost healthcare charges for the EP cohort were 5.4 and 2.3 times higher than for the M-LP and VP cohorts, respectively. Findings were similar for respiratory-related healthcare charges; unadjusted ratios of healthcare charges were 10.3 and 3.5 times higher, respectively. There were no differences between cohorts in healthcare charge cost ratios when demographic characteristics and comorbidities were considered (adjusted ratios of 1.2 for both comparisons); however, for respiratory-related healthcare charges, the adjusted ratios were 1.6 and 1.4 times higher than the EP cohort compared with the ML-P and VP cohorts, respectively.

Societal costs showed a similar pattern as healthcare charges; all-cause total societal costs were USD $28 504, $21 723, and $15 892 for EP, VP, and M-LP infants, respectively (Figure 3B). Respiratory-related societal costs in the cohorts were USD $7528, $5238, and $3856, respectively.

## DISCUSSION

In this study, we evaluated the effect of GA on the prevalence of comorbidities, HCRU, and direct healthcare charges and societal costs associated with preterm birth using birth claims and follow-up claims up to 2 years CA from a large public insurance system in the United States (Medicaid). We found considerable increases in the number of comorbidities, rehospitalizations, ED visits, outpatient visits, direct charges, and societal costs associated with EP infants vs those born at more than 28 wGA. Respiratory comorbidities were a significant driver of the cost increases.

The underdeveloped respiratory system is a major contributor to morbidity and mortality in premature infants. The findings from our analysis underscore the costs associated with respiratory comorbidities such as BPD and the need for improvement in respiratory care to minimize the risk for development of these complications. Although protocols and standard of care vary between healthcare systems in the United States, some quality improvement networks (ie, collaborations within states) have developed more specific guidelines for respiratory support in the EP population. Standardizing care has long been shown to improve outcomes and therefore would be a valuable practice to start toward decreasing the incidence of BPD and its associated long-term consequences.

From our analysis of 25 573 infants born prematurely in 6 US state Medicaid databases from 1997 to 2018, 17.4% were EP (<28 wGA). Of note, the proportion of infants who were Black increased with lower GA, whereas the proportion of White infants increased with higher GA. The proportion of EP infants overall in our study was higher than the previous reported national estimate of 6%.^3^ The higher proportion of EP births we found in our data may be related to the low socioeconomic status of Medicaid insurance recipients in the 6 states analyzed. Socioeconomic factors of preterm birth in the United States have been documented^44^; in the present analysis, 5 of the 6 states included are ranked in the lower half of all US states by median household income (2015).^45^

Birth hospitalization LOS was found to approximately double with each 4-week decrease in GA in our study. This is in agreement with a 2006-2007 study of preterm birth in Canada, in which LOS was 83.1 days for infants born at less than 28 wGA, 42.6 days for infants 28 to 31 wGA, and 21.2 days for infants 32 to 33 wGA.^22^ Similarly, in a UK study of preterm births 24 to 31 wGA, LOS ranged from 123 days for infants 24 wGA to 66 days for infants 28 wGA.^46^ Mean follow-up times were 8% (19 days) longer for infants born at 28 or more wGA, which may be due to increased mortality for EP infants, but these data were not available for confirmation.

In our study, RDS was the most common comorbidity across all GA cohorts, affecting 57.5% of infants; twice as many infants in the EP cohort were affected (91.5%) than in the M-LP cohort (45.3%). This is consistent with recent reviews on complications of prematurity that find respiratory complications among the most common comorbidity in preterm infants.^7,30,31,44^ In a retrospective study of 4292 preterm births at 24 to 36 wGA at the Medical University of South Carolina, RDS was the most common comorbidity, affecting 19.8% of infants.^15^ Respiratory distress syndrome was reported to increase with decreasing GA in a study of 147 224 singleton preterm births, affecting 58.5% of infants born at 28 wGA compared with 82.3% of infants born at 25 wGA.^18^

Bronchopulmonary dysplasia, the next most frequently occurring comorbidity across all GA cohorts in the present study, affected 50.6% of EP infants; the prevalence was 3.5 times higher than in the VP cohort (14.4%) and more than 20 times higher in the EP cohort than in the M-LP cohort (2.4%). Invasive respiratory support is a major contributor to the development of BPD^47^; thus, its prevalence is intrinsically linked with overall respiratory complications. In EPIPAGE-2, a national, prospective, population-based cohort study conducted in all maternity and neonatal units in France, severe BPD was reported in 25.6% of infants born at 23 to 26 wGA, 4.6% of infants 27 to 31 wGA, and 0% of infants 32 or more wGA.^34^ The trend of increased prevalence of PDA, IVH, ROP, and PVL with decreasing GA, observed to varying degrees in this study, is also in agreement with several recent studies.^15,34,48^

In the present study, all-cause rehospitalizations were reported in just under half of EP infants (44.8%), compared with a quarter of ML-P infants. For EP infants with BPD, this percentage rose to 51.6%. These findings are higher overall than reported in previous studies, although the trends by GA are consistent. The rates of rehospitalization found by Reed et al^49^ in EPIPAGE-2 are 15.2% for preterm infants born at 22 to 26 wGA who were rehospitalized, compared with 10.1% of those 27 to 31 wGA and 4.0% of those 32 to 34 wGA. In a retrospective cohort study of infants born at less than 33 wGA, Smith et al reported a rehospitalization rate of 22.7% overall, which rose to 49.6% for infants with BPD at initial discharge.^50^

Our study found that in the first 2 years of life, total healthcare charges for EP infants were significantly higher than for infants born at more than 28 wGA; total all-cause healthcare charges in USD, inflated to 2018 values, were 5.4 times higher in the EP cohort ($843 499) than in the M-LP cohort ($158 430), and 2.3 times higher than in the VP cohort ($379 581). Excluding birth hospitalization, charges associated with the treatment of respiratory comorbidities accounted for 52.7% of the all-cause costs for the EP cohort, vs 43.5% for the M-LP cohort. Healthcare charges have been found to increase with decreasing GA in numerous previous studies.^10,14–17,19–23,27,28,50,51^ A Canadian study by Bérard et al^14^ found that the mean total healthcare costs in the first 2 years of life are almost double for late preterm births compared with full-term births (CAD $2568 preterm vs CAD $1285 full-term; cost ratio, 1.99 [95% confidence interval, 1.90-2.09]); however, data did not address lower GA cohorts. Increasing healthcare costs are also associated with increasing rates of comorbidities as GA decreases.^15,24,48^ Black et al^15^ modeled the cost of individual comorbidities of preterm births and calculated the increase with each week reduction in GA. Healthcare charges associated with treatment of BPD were estimated at USD $23 132 for an infant born at 24 wGA compared with USD $9356 for an infant born at 36 wGA.

We found that all-cause societal costs increased 1.8-fold for EP infants compared with those born M-LP. However, there is a paucity of reported data comparing societal costs of preterm birth by GA. The Neonatal Adequate Care for Quality of Life study of infants up to age 18 months compared very low birthweight infants without prematurity-related morbidities with full-term infants to assess productivity loss costs during the initial hospitalization.^16^ These costs were estimated to be more than 5 times higher for very low birthweight vs full-term infants, and total societal costs of initial hospitalization from birth to discharge were more than 12 times higher.^16^

### Limitations

Although our findings are in line with previous studies on the health burden and healthcare costs of preterm birth, there are limitations to the current study, including limitations related to the nature of data collected from medical insurance claims. The use of ICD-9-CM and ICD-10-CM diagnosis codes for medical claims approximate actual diagnoses and may not capture all relevant medical diagnoses. Medical services obtained outside the Medicaid system are not captured. Interstate differences in the coding of Medicaid patient data require a non-uniform treatment of the study data. For example, dates of birth of infants from New Jersey and Missouri were not available, due to requirements for de-identification of data; in these instances, the date of birth was approximated using the date of the earliest claim in the birth year. For premature infants identified by ICD-9-CM and ICD-10-CM diagnosis codes, GA was conservatively approximated using the upper bound of the GA range. Because the Medicaid system is a public insurance with strict maximum income eligibility requirements, our results may not be representative of data from private insurers or a broader population.

Limitations of our analytic methods include a variable follow-up period across infants, due to a small number of infants who died before 2 years CA. Additionally, there was a high prevalence of capitation claims throughout the dataset; thus, actual healthcare costs could not be estimated without strong assumptions. Estimates of work time lost (due to ED and outpatient visits) are reflective of the geographic regions analyzed, and could be considered comparatively low, resulting in underestimation of total societal costs. Lastly, premature infants included in this study were identified retrospectively from 1997 to 2018, and it is possible that clinical care changes over that period may have affected the results.

## CONCLUSIONS

This retrospective cohort analysis of US Medicaid data demonstrated that presence of comorbidities, HCRU, direct hospital charges, and societal costs from birth to 2 years CA were higher among EP infants than for premature infants born at more than 28 wGA and increased with decreasing GA. A significant portion of healthcare costs is associated with respiratory complications of preterm birth, which increase in prevalence with decreasing GA. Our findings highlight a need for strategies to improve clinical outcomes for EP infants to reduce the burden of comorbidities of prematurity on healthcare systems and families.

### Author Contributions

M.E.M. and S.P.S. contributed to the conception and design, interpretation of data, and drafting/revising of the article critically for important intellectual content. W.G., H.S., P.Z., H.W., and R.A. contributed to the acquisition of data and analysis, interpretation of data, and drafting/revising of the article critically for important intellectual content. All authors approved the final manuscript.

### Disclosures

M.E.M. declares no competing interests. W.G., H.S., P.Z., H.W., and R.A. are employees of Analysis Group, Inc., which was contracted through Shire, a Takeda company, to perform this study. S.P.S. was an employee of Shire, a Takeda company, at the time of the study.

### Data Availability

The data that support the findings of this study are available from Medicaid records from individual states, but restrictions apply to the availability of these data due to applicable privacy laws and data use agreements. The data were used pursuant to data use agreements between Analysis Group, Inc. and the individual states. Requests for data access should be directed to Alexandra Miller, Data Governance, Analysis Group, Inc. (Alexandra.Miller@analysisgroup.com). The data may be available with permission directly from individual states, subject to the state’s requirements for data access to Medicaid records.

## Supplementary Material

Online Supplementary Material
