# Supplementary material for: Burden of Comorbidities and Healthcare Resource Utilization Among Medicaid-Enrolled Extremely Premature Infants
Source: J Health Econ Outcomes Res. 2022 Dec 23;9(2):147–55. doi: 10.36469/001c.38847 (PMC9790150; doi:10.36469/001c.38847)
Supplement: Online Supplementary Material [file jheor_2022_9_2_38847_122372.pdf]

Journal of Health Economics and Outcomes Research

Online Supplementary Material

Burden of Comorbidities and Healthcare Resource Utilization Among Medicaid-Enrolled Extremely Premature Infants. *JHEOR*. **2022;9(2):147-155.** doi:10.36469/jheor.2022.38847

Table S1: ICD-9-CM and ICD-10-CM Codes to Identify Premature Infants

Table S2: Hospital Revenue Codes to Identify NICU/ICU Admissions

Table S3: ICD-9-CM and ICD-10-CM Codes to Identify Congenital Heart Disease, Diaphragmatic Hernia, and Congenital Malformations

Table S4: ICD-9-CM and ICD-10-CM Codes to Identify Respiratory Causes of Hospitalization, Outpatient, or Emergency Department Visits

Table S5: GPI Codes to Identify Respiratory Medication Uses

Table S6: HCPCS Codes Used to Identify Home Oxygen Therapy/Respiratory Aids

Table S7: ICD-9-CM and ICD-10-CM Codes Used to Identify Comorbidities

Table S8: ICD-9, ICD-10, CPT, and HCPCS Codes of Common Procedures Associated With Prematurity Comorbidities

Table S9: ICD-9-CM, ICD-10-CM Codes of Delivery Methods

This supplementary material has been provided by the authors to give readers additional information about their work.

This is an open-access article distributed under the terms of the Creative Commons Attribution 4.0 International License (CCBY-4.0). View this license's legal deed at http://creativecommons.org/licenses/by/4.0 and legal code at http://creativecommons.org/licenses/by/4.0/legalcode for more information.

Table S1. ICD-9-CM and ICD-10-CM Codes to Identify Premature Infants

Code Version	Diagnosis Code	Description
ICD-9-CM	765.21	Less than 24 completed weeks of gestation
	765.22	24 completed weeks of gestation
	765.23	25-26 completed weeks of gestation
	765.24	27-28 completed weeks of gestation
	765.25	29-30 completed weeks of gestation
	765.26	31-32 completed weeks of gestation
	765.27	33-34 completed weeks of gestation
	765.28	35-36 completed weeks of gestation
ICD-10-CM	P07.21	Extreme immaturity of newborn, gestational age less than 23 completed weeks
	P07.22	Extreme immaturity of newborn, gestational age 23 completed weeks
	P07.23	Extreme immaturity of newborn, gestational age 24 completed weeks
	P07.24	Extreme immaturity of newborn, gestational age 25 completed weeks
	P07.25	Extreme immaturity of newborn, gestational age 26 completed weeks
	P07.26	Extreme immaturity of newborn, gestational age 27 completed weeks
	P07.31	Preterm newborn, gestational age 28 completed weeks
	P07.32	Preterm newborn, gestational age 29 completed weeks
	P07.33	Preterm newborn, gestational age 30 completed weeks
	P07.34	Preterm newborn, gestational age 31 completed weeks
	P07.35	Preterm newborn, gestational age 32 completed weeks
	P07.36	Preterm newborn, gestational age 33 completed weeks
	P07.37	Preterm newborn, gestational age 34 completed weeks
	P07.38	Preterm newborn, gestational age 35 completed weeks
	P07.39	Preterm newborn, gestational age 36 completed weeks

Table S2. Hospital Revenue Codes to Identify NICU/ICU Admissions

Admission Unit	Revenue Code	Description
NICU	174	Nursery - newborn - level IV (intensive care)
	175	Nursery - neonatal ICU
ICU	200	Intensive care - general classification
	201	Intensive care - surgical
	202	Intensive care - medical
	203	Intensive care - pediatric
	206	Intensive care – post ICU or intermediate ICU
	209	Intensive care - other intensive care

Abbreviations: ICU, intensive care unit; NICU, neonatal intensive care unit.

Table S3. ICD-9-CM and ICD-10-CM Codes to Identify Congenital Heart Disease, Diaphragmatic Hernia, and Congenital Malformations

Comorbidities		ICD-9-CM Code(s)	ICD-10-CM Code(s)
Congenital heart diseases	Bulbus cordis anomalies and anomalies of cardiac septal closure (excluding patent foramen ovale)	745.1-745.4, 745.6-745.9	Q20, Q21.0, Q21.2-Q21.9
	Other congenital anomalies of heart	746	Q22, Q23, Q24
	Other congenital anomalies of circulatory system (excluding patent ductus arteriosus)	747.1-747.9	Q25.1-Q25.9, Q26, Q27, Q28
Diaphragmatic hernia		756.6	Q79.0, Q79.1
Congenital malformations	Other congenital musculoskeletal deformities of chest	754.89	Q67.8
	Congenital malformations of lung	748.4-748.6	Q33
	Congenital malformations of upper airway	748.0-748.3	Q30, Q31, Q32

Table S4. ICD-9-CM and ICD-10-CM Codes to Identify Respiratory Causes of Hospitalization, Outpatient, or Emergency Department Visits

Comorbidity	ICD-9-CM Code(s)	ICD-10-CM Code(s)
Acute lower respiratory infection	—	J22
Acute respiratory distress	518.82	J80
Acute respiratory distress during index hospitalization	518.82, 769	J80, P22
Apnea	786.03	R06.81
Asthma	493, V17.5	J45, Z82.5
Bronchitis	466.0, 490, 491	J20.0-J20.9, J22, J40
Bronchiolitis	466.1	J21
Cough	786.2	R05
Cyanosis	782.5	R23.0
Dyspnea	786	R06.00, R06.01, R06.02, R06.09, R06.3, R06.4, R06.81, R06.82, R06.83, R06.89, R06.9
Hypoxemia	770.88, 799.02	R09.02, P84
Hypoxia	768	J96.01, J96.11, J96.21, J96.91
Pneumonia/influenza	480-488	J09-J18
Reactive airway disease	519.8, 493.9	J98.8, J45.901, J45.902, J45.909, J45.998
Respiratory failure	518.81	J96.00, J96.90
Respiratory syncytial virus	79.6	B97.4
Stridor	786.1	R06.1
Tachypnea	786.06	R06.82
Tracheostomy complications	519	J95.0
Upper respiratory tract infection	465.8, 465.9	J00-J06
Wheeze	786.07	R06.2

Table S5. GPI Codes to Identify Respiratory Medication Uses

Category	Drug	HCPCS	GPI Code
Humanized monoclonal antibody	Palivizumab	S9562	1950206000
Inhaled bronchodilator	Albuterol	J7602, J7603, J7609, J7610, J7611, J7613, J7616, J7618, J7619, J7620, J7621, Q4093, Q4094	4420101000
			4420101010
			4420990201
			4420990202
			4420990280
	Ipratropium bromide	J7644, J7645	4230004010
			4410003010
			4410003012
	Levalbuterol	J7607, J7612, J7614, J7615, J7617	4420104510
			4420104550
			9664506560
			9664506570
	Racemic epinephrine	N/A	8840077010
			8840990250
Methylxanthine	Aminophylline	J0280	4430001000
			4430001001
			4430001010
			4430001012
	Caffeine citrate	J0706	6130001010

Table S5. GPI Codes to Identify Respiratory Medication Uses

Category	Drug	HCPCS	GPI Code
Methylxanthine	Theophylline	J2810	4430004000
			4430004001
			4430004010
			4430004020
			4499880270
			9630006700
Inhaled steroid	Beclomethasone	J7622	4220001030
			4220001032
			4440001010
			9644500912
			2210000610
			4420990230
			4420990202
	Budesonide	J7626, J7627, J7633, J7634	4220001500
			4440001500
			2210001200
			4420990240
			4420990241
			9644821200
	Ciclesonide	N/A	4220001800
			4440001700
	Flunisolide	J7641	4220003000
			4440003000
			4440003012
	Fluticasone	N/A	4220003210
			4220003230
			4250990224
			4440003320
			9652646520
			9055006810
			4440003322
			4440003310
			4420990270
	Mometasone	S1090	4220004510
			9666704020
			4440003620
			4420990290
	Triamcinolone	J7683, J7684	4440004020
			9680762703
			9680762705
			4220006010
			2210005000
			2210005010
			2210005020
			2210005022

Table S5. GPI Codes to Identify Respiratory Medication Uses

Category	Drug	HCPCS	GPI Code
Inhaled steroid	Dexamethasone	J7637, J7638	4440002010
			4220002010
Pulmonary vasodilator	Bosentan	N/A	4016001500
	Epoprostenol sodium	J1325	4017004010
	Iloprost tromethamine	Q4074	4017006000
			4017006020
	Sildenafil	S0090	4014306010
			4030407000
			4030407010
			9678583412
	Treprostinil	J3285, J7686	4017008000
			4017008005
			4017008010
Diuretic	Bumetanide	S0171	3720001000
			3799100220
	Chlorothiazide	J1205	3760002000
			9646563742
			3760002010
	Furosemide	J1940	3720003000
			3720003020
			3799000217
			3799100235
	Hydrochlorothiazide	N/A	3760004000
			3799000220
	Metolazone	N/A	3760006000
	Spironolactone	N/A	3750002000
			3799000220
			3799000217
			3799000214
	Bendroflumethiazide	N/A	3760001000
	Benzthiazide	N/A	3760001500
	Chlorthalidone	N/A	3760002500
	Cyclopenthiazide	N/A	3760002800
	Cyclothiazide	N/A	3760003000
	Flumethiazide	N/A	3760003500
	Hydroflumethiazide	N/A	3760004500
			3799000224
			9656881150
	Indapamide	N/A	3760005000
	Mefruside	N/A	3760005300
	Methyclothiazide	N/A	3760005500
	Metolazone	N/A	3760006000
	Polythiazide	N/A	3760006500
	Quinethazone	N/A	3760007000
	Trichlormethiazide	N/A	3760007500
			9680762710

Table S5. GPI Codes to Identify Respiratory Medication Uses

Category	Drug	HCPCS	GPI Code
Diuretic	Bendroflumethiazide with KCl	N/A	3799100210
	Hydrochlorothiazide-KCl	N/A	3799100250
	Acetazolamide	J1120	9642461565
			3710001000
			3710001010
Systemic corticosteroid	Hydrocortisone	J1700, J1710, J1720	2210002500
			2210002510
			2210002520
			2210002525
			2210002530
			2210002540
	Methylprednisolone	J1020, J1030, J1040, J2920, J2930, J7509	2210003000
			2210003010
			2210003015
			9666507065
			8630004510
			2210003020
	Prednisone/ prednisolone	J7506, J7512, J2650, J7510	2210004500
			2210004000
			2210004010
			2210004020
			2210004025
			2210004030
			2210990220
	Dexamethasone	J1100, C9256, J7312, J1094, J8540	2210002000
			2210002010
			2210002015
			9648507080
			2210002017
			2210002020
			2210002030
			2210990215
			2210002021
			2210990255
			2210990250
Leukotriene receptor antagonist (LTRA)	Montelukast	N/A	4450505010
			9666704470
Bronchodilator	Terbutaline	J3105, J7680, J7681	4420106020
			4499400370
			4499400270

Table S6. HCPCS Codes Used to Identify Home Oxygen Therapy/Respiratory Aids

HCPCS Code	Description
E1390	Oxygen concentrator, single delivery port
E1391	Oxygen concentrator, dual delivery port
E0424	Stationary compressed gaseous oxygen system, rental; includes container, contents, regulator, flowmeter, humidifier, nebulizer, cannula or mask, and tubing
E0439	Stationary liquid oxygen system, rental; includes container, contents, regulator, flowmeter, humidifier, nebulizer, cannula or mask, and tubing
E1405	Oxygen and water vapor enriching system with heated delivery
E1406	Oxygen and water vapor enriching system without heated delivery
E0431	Portable gaseous oxygen system, rental; includes portable container, regulator, flowmeter, humidifier, cannula or mask, and tubing
E0434	Portable liquid oxygen system, rental; includes portable container, supply reservoir, humidifier, flowmeter, refill adaptor, contents gauge, cannula or mask, and tubing
E1392	Portable oxygen concentrator, rental
E0433	Portable liquid oxygen system, rental; home liquefier used to fill portable liquid oxygen containers, includes portable containers, regulator, flowmeter, humidifier, cannula or mask and tubing, with or without supply reservoir and contents gauge
K0738	Portable gaseous oxygen system, rental; home compressor used to fill portable oxygen cylinders; includes portable containers, regulator, flowmeter, humidifier, cannula or mask, and tubing
E0441	Stationary oxygen contents, gaseous, 1 month's supply = 1 unit
E0442	Stationary oxygen contents, liquid, 1 month's supply = 1 unit
E0443	Portable oxygen contents, gaseous, 1 month's supply = 1 unit
E0444	Portable oxygen contents, liquid, 1 month's supply = 1 unit

Table S7. ICD-9-CM and ICD-10-CM Codes Used to Identify Comorbidities

**Comorbidities**	**ICD-9-CM Codes**	**ICD-10-CM Codes**
Bacterial meningitis	320	G00, G01
Bacterial sepsis (early or late-onset)	038.19, 771.81, 995.91, 995.92	A40, A41, P36, R65.2
Bronchopulmonary dysplasia	496, 518.89, 770.7	P27.1, J44.9, J98.4
General neurological dysfunction	779.0-779.2	P90, P91
Hearing loss	389, 744.0	H90, H91
Intracerebral hemorrhage	431, 767	I61, P52.4
Intraventricular hemorrhage	772.1	P52.0-P52.3
Stage 1	772.11	P52.0
Stage 2	772.12	P52.1
Stage 3	772.13	P52.21
Stage 4	772.14	P52.22
Unspecified stage	772.1	P52.3
Necrotizing enterocolitis	777.5	P77
Stage 1	777.51	P77.1
Stage 2	777.52	P77.2
Stage 3	777.53	P77.3
Unspecified stage	777.5	P77.9
Patent ductus arteriosus	747	Q25.0
Periventricular leukomalacia	779.7	P91.2
Retinal detachment	361, 362.26, 362.27	H33.0, H35.159, H35.169
Retinopathy of prematurity	362.2	H35.1 except H35.11
Stage 1	362.23	H35.12
Stage 2	362.24	H35.13
Stage 3	362.25	H35.14
Stage 4	362.26	H35.15
Stage 5	362.27	H35.16
Unspecified stage	362.20	H35.10
Spontaneous intestinal perforation	569.83	K63.1

Table S8. ICD-9, ICD-10, CPT, and HCPCS Codes of Common Procedures Associated With Prematurity Comorbidities

Procedure	ICD-9 Codes	ICD-10 Codes	CPT/HCPCS
Bowel surgery for NEC	45.61, 45.62, 45.7, 45.71, 45.72, 45.73, 45.74, 45.75, 45.76, 45.79, 45.8, 46.0, 46.01, 46.03, 46.1, 46.10, 46.11, 46.13, 46.2, 46.20, 46.21, 46.23, 46.39	0DT80ZZ, 0DT84ZZ, 0DT87ZZ, 0DT88ZZ, 0DTE0ZZ, 0DTE4ZZ, 0DTE7ZZ, 0DTE8ZZ, 0DTF0ZZ, 0DTF4ZZ, 0DTF7ZZ, 0DTF8ZZ, 0DTG0ZZ, 0DTG4ZZ, 0DTG7ZZ, 0DTG8ZZ, 0DTGFZZ, 0DBE0ZX, 0DBE0ZZ, 0DBE3ZX, 0DBE3ZZ, 0DBE4ZX, 0DBE4ZZ, 0DBE7ZX, 0DBE7ZZ, 0DBE8ZX, 0DBE8ZZ, 0DBF0ZX, 0DBF0ZZ, 0DBF3ZX, 0DBF3ZZ, 0DBF4ZX, 0DBF4ZZ, 0DBF7ZX, 0DBF7ZZ, 0DBF8ZX, 0DBF8ZZ	44140, 44141, 44143, 44144, 44145, 44146, 44147, 44150, 44151, 44155, 44156,44157, 44158, 44160, 50810, 57307
Hydrocephalus shunting for IVH	V45.2	Z98.2	62220, 62223, 62190, 62192, 62194, 62225, 62230, 62252, 62180, 62200, 62201
Intubation for BPD/RDS	96	0BH17EZ, 0BH18EZ, 0BH13EZ	31500, 43752, 91105, 93503
Laser surgery/cryotherapy for ROP	NA	NA	67228, 67229, J9035
Patent ductus arteriosus ligation	NA	NA	33820
Tracheostomy for BPD/RDS	31.1, 31.2, 31.41, 31.74, 33.21, 96.55	Z93.0, J95.0, 0B21XFZ	31600, 31601, 31603, 31605, 31610, A4626, A4629, A7523, A7525, A7526, L8501

Abbreviations: BPD, bronchopulmonary dysplasia; IVH, intraventricular hemorrhage; NEC, necrotizing enterocolitis; RDS, respiratory distress syndrome; ROP, retinopathy of prematurity.

Table S9. ICD-9-CM, ICD-10-CM Codes of Delivery Methods

Procedures	ICD-9-CM Codes	ICD-10-CM Codes
Single liveborn infants, delivered vaginally or outside hospital	V30.00, V30.1, V30.2, V39.00, V39.1, V39.2	Z38.00, Z38.1, Z38.2
Single liveborn infants, delivered by cesarean	V30.01, V39.01	Z38.01
Twin liveborn infants, delivered vaginally or outside hospital	V31.00, V31.1, V31.2, V32.00, V32.1, V32.2, V33.00, V33.1, V33.2	Z38.30, Z38.4, Z38.5
Twin liveborn infants, delivered by cesarean	V31.01, V32.01, V33.01	Z38.31
Multiple liveborn infants, delivered vaginally or outside hospital	V34.00, V34.1, V34.2, V35.1, V35.2, V36.1, V36.2, V37.00, V37.1, V37.2, V36.00	Z38.61, Z38.63, Z38.65, Z38.68, Z38.7, Z38.8
Multiple liveborn infants, delivered by cesarean	V34.01,V35.00, V35.01, V36.00, V36.01, V37.01	Z38.62, Z38.64, Z38.66, Z38.69
